# AGE-RAGE Axis Stimulates Oxidized LDL Uptake into Macrophages through Cyclin-Dependent Kinase 5-CD36 Pathway via Oxidative Stress Generation

**DOI:** 10.3390/ijms21239263

**Published:** 2020-12-04

**Authors:** Hironori Yashima, Michishige Terasaki, Ami Sotokawauchi, Takanori Matsui, Yusaku Mori, Tomomi Saito, Naoya Osaka, Hideki Kushima, Munenori Hiromura, Makoto Ohara, Tomoyasu Fukui, Sho-ichi Yamagishi

**Affiliations:** 1 Department of Medicine, Division of Diabetes, Metabolism, and Endocrinology, Showa University School of Medicine, Tokyo 142-8666, Japan; yashima@med.showa-u.ac.jp (H.Y.); saito_to@cnt.showa-u.ac.jp (T.S.); n.oosaka0709@gmail.com (N.O.); hkushima@med.showa-u.ac.jp (H.K.); hiromura@med.showa-u.ac.jp (M.H.); s6018@nms.ac.jp (M.O.); showauft@med.showa-u.ac.jp (T.F.); shoichi@med.showa-u.ac.jp (S.-i.Y.); 2 Department of Pathophysiology and Therapeutics of Diabetic Vascular Complications, Kurume University School of Medicine, Kurume 830-0011, Japan; sotokawauchi_ami@med.kurume-u.ac.jp (A.S.); matsui_takanori@med.kurume-u.ac.jp (T.M.); 3 Anti-Glycation Research Section, Department of Medicine, Division of Diabetes, Metabolism, and Endocrinology, Showa University School of Medicine, Tokyo 142-8666, Japan; u-mori@med.showa-u.ac.jp

**Keywords:** AGEs, Cdk5, CD36, RAGE-aptamer, macrophages

## Abstract

Advanced glycation end products (AGEs) are localized in macrophage-derived foam cells within atherosclerotic lesions, which could be associated with the increased risk of atherosclerotic cardiovascular disease under diabetic conditions. Although foam cell formation of macrophages has been shown to be enhanced by AGEs, the underlying molecular mechanism remains unclear. Since cyclin-dependent kinase 5 (Cdk5) is reported to modulate inflammatory responses in macrophages, we investigated whether Cdk5 could be involved in AGE-induced *CD36* gene expression and foam cell formation of macrophages. AGEs significantly increased Dil-oxidized low-density lipoprotein (ox-LDL) uptake, and *Cdk5* and *CD36* gene expression in U937 human macrophages, all of which were inhibited by DNA aptamer raised against RAGE (RAGE-aptamer). *Cdk5* and *CD36* gene expression levels were correlated with each other. An antioxidant, *N*-acetyl-l-cysteine, mimicked the effects of RAGE-aptamer on AGE-exposed U937 cells. A selective inhibitor of Cdk5, (R)-DRF053, attenuated the AGE-induced Dil-ox-LDL uptake and *CD36* gene expression, whereas anti-CD36 antibody inhibited the Dil-ox-LDL uptake but not *Cdk5* gene expression. The present study suggests that AGEs may stimulate ox-LDL uptake into macrophages through the Cdk5–CD36 pathway via RAGE-mediated oxidative stress.

## 1. Introduction

Diabetes increases the risk of atherosclerotic cardiovascular disease, and half of diabetic patients die from cardiovascular disorders [1]. According to the recent report of the International Diabetes Federation (IDF) Diabetes Atlas in 2019, the number of diabetes patients aged 20–79 is estimated to be 463 million worldwide. Macrovascular complications, such as coronary artery disease, peripheral artery disease, and stroke, could account for the comorbidities and increased risk of death in patients with diabetes [1]. As a result, the average lifespan is decreased by more than 20 years in middle-aged type 1 diabetes patients and by nearly 10 years in middle-aged type 2 diabetes patients in comparison with that of non-diabetic subjects [2].

Various biochemical pathways activated under diabetic conditions could contribute to the acceleration of atherosclerosis [3,4]. Among them, advanced glycation end products (AGEs), the formation and accumulation of which are enhanced under diabetic conditions, are considered to play a crucial role in the development and progression of atherosclerotic cardiovascular disease [5,6,7]. Nonenzymatic glycation and cross-linking of extracellular matrix proteins alter their structural integrity and functional properties, thereby contributing to the increased vascular and myocardial stiffness involved in cardiovascular disease [8]. The interaction of AGEs with a cell surface receptor of AGE (RAGE), which belongs to the immunoglobulin superfamily, has been shown to evoke oxidative stress, as well as inflammatory and thrombotic reactions, resulting in the progression of atherosclerosis [9]. The engagement of RAGE with AGEs could promote the formation and accumulation of AGEs and induce RAGE expression via oxidative stress generation, thereby creating a positive feedback loop between AGEs and the RAGE downstream pathway in numerous types of cells and tissues [10]. AGEs are localized in macrophage-derived foam cells within the atherosclerotic lesions, which may be involved in atherosclerotic plaque instability and the increased risk of atherosclerotic cardiovascular disease in patients with diabetes mellitus [11,12,13,14,15]. We have previously found that continuous infusion of DNA-aptamer raised against RAGE (RAGE-aptamer) significantly inhibits the development and progression of experimental diabetic nephropathy, attenuates the growth and metastasis of melanoma in nude mice, and prevents renal damage in hypertensive mice [16,17,18,19]. These findings support the clinical relevance of RAGE-aptamer in various devastating AGE-related disorders. Therefore, inhibition of the AGE-RAGE axis by RAGE-aptamer may also be a potential therapeutic target against acceleration of atherosclerosis in diabetes mellitus.

The accumulation of cholesterol esters and foam cell formation of macrophages are among the earliest characteristic features of atherosclerosis [3,4]. Low-density lipoprotein (LDL) is subject to oxidative modifications in the subendothelial space to become oxidized LDL (ox-LDL). ox-LDL stimulates expression of cell adhesion molecules and chemokines, inducing monocyte adhesion to endothelial cells and subsequent migration into the subendothelial space [3,20]. During this process, monocytes are differentiated to macrophages, and ox-LDL uptake by macrophages is partly dependent on scavenger receptor CD36, which can stimulate the foam cell formation of macrophages within the atherosclerotic plaques [3,20]. Under diabetic conditions, foam cell formation and the related *CD36* gene expression of macrophages have been known to be enhanced, contributing to the accelerated atherosclerosis in diabetes mellitus [21,22,23,24,25]. We have previously reported that foam cell formation and *CD36* expression are significantly increased in monocyte-derived macrophages isolated from patients with type 1 and type 2 diabetes compared with those from nondiabetic healthy controls [21,26]. We recently found that AGEs significantly increase ox-LDL uptake and *CD36* gene expression in human cultured macrophages [26]. However, the underlying molecular mechanism for accelerated foam cell formation of macrophages in diabetes remains unclear. In other words, how AGEs stimulate the foam cell formation of macrophages is not fully understood.

Cyclin-dependent kinases (Cdks) play essential roles in cell cycle regulation, apoptosis, transcription, and differentiation [27,28]. Among them, proline-directed serine/threonine cyclin-dependent kinase 5 (Cdk5) is a unique Cdk family member; in contrast to other Cdk members, Cdk5 is not a regulator of cell cycle progression [29,30,31] but a modulator of gene regulation, cell survival, and apoptosis [32]. Cdk5 was first identified as a neuronal cdc2-like kinase with 58% and 61% amino acid sequence homology to mouse Cdk1 and human Cdk2, respectively [32,33]. Cdk5 can phosphorylate the lysine-serine-proline motif of neurofilaments, thereby playing a role in neuronal cell development, migration, and differentiation, whereas its functional deterioration is associated with Alzheimer’s disease [32,33,34]. Recently, Cdk5 has been shown to contribute to endothelial cell senescence and tumor angiogenesis as well [29,35]. A truncated regulatory subunit of Cdk5 is accumulated in the atherosclerotic areas of aortas, whereas the inhibition of Cdk5 not only attenuates the expression of inflammatory genes in endothelial cells, but also suppresses the development of atherosclerosis in apolipoprotein E-deficient mice [29]. In addition, Cdk5 is constitutively expressed in macrophages and could contribute to inflammatory reactions in lipopolysaccharide-stimulated macrophages [30]. These observations led us to speculate that Cdk5 could be a modulator of foam cell formation in AGE-exposed macrophages and that the inhibition of Cdk5 in macrophages may have favorable effects on foam cell formation of macrophages. Thus, we examined here whether and how Cdk5 is involved in AGE-induced *CD36* gene expression and foam cell formation of macrophages using various types of inhibitors for the AGE-signaling pathway.

## 2. Results

### 2.1. RAGE-Aptamer Inhibited the AGE-Induced Dil-ox-LDL Uptake, and Cdk5 and CD36 Gene Expression in U937 Cells

**Figure 1:**
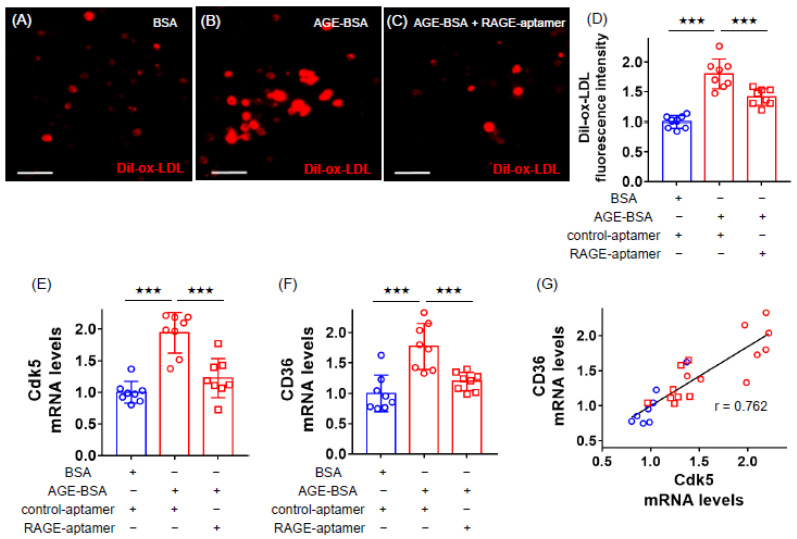
Effects of RAGE-aptamer on Dil-ox-LDL uptake, *Cdk5* and *CD36* gene expression in advanced glycation end product (AGE)-exposed U937 cells. U937 macrophages were treated with 100 µg/mL AGE-bovine serum albumin (AGE-BSA), 100 µg/mL non-glycated BSA in the presence or absence of 100 nmol/L RAGE-aptamer, or 100 nmol/L control-aptamer in Roswell Park Memorial Institute (RPMI) 1640 medium supplemented with 10% fetal bovine serum including 100 µg/mL streptomycin and 100 U/mL penicillin at 37 °C in 5% CO_2_ for 24 h. The cells then were incubated with 10 μg/mL Dil-ox-LDL in the same RPMI 1640 medium for 18 h to evaluate the fluorescence intensity. (**A**–**C**) Representative immunofluorescent staining images. Dil-ox-LDL-positive cells were stained in red. Scale bars represent 50 µm. (**D**) Quantitative data of fluorescence intensity. Dil-ox-LDL uptake was normalized by the control values with BSA. (**E**–**G**) Gene expression levels of *Cdk5* (**E**) and *CD36* (**F**) and their correlation (**G**). Total RNAs were transcribed and amplified by real-time PCR. Data were normalized by the intensity of *GAPDH* mRNA-derived signals and then related to the control values with BSA. The correlation between *Cdk5* and *CD36* gene expression levels was determined by Pearson’s correlation test. Number = 8 for each group. Results are presented as mean ± standard deviation. ^★★★^ *p* < 0.005 vs. treatment of AGE-BSA.

We first examined the effects of AGEs on ox-LDL uptake, and *Cdk5* and *CD36* gene expression in U937 macrophages. As shown in Figure 1A–D, compared with non-glycated bovine serum albumin (BSA), AGEs remarkably increased ox-LDL uptake into macrophages evaluated by 1,1′-dioctadecyl-3,3,3′,3′-tetramethylindocarbocyanine perchlorate (Dil)-ox-LDL immunofluorescent staining, which was significantly inhibited by the treatment with RAGE-aptamer. AGE-BSA significantly up-regulated *Cdk5* and *CD36* mRNA levels in U937 cells, both of which were attenuated by RAGE-aptamer (Figure 1E,F). *Cdk5* and *CD36* gene expression levels were correlated with each other (Figure 1G).

### 2.2. Effects of NAC, (R)-DRF053 and Anti-CD36 Antibody on AGE-Exposed U937 Cells

**Figure 2:**
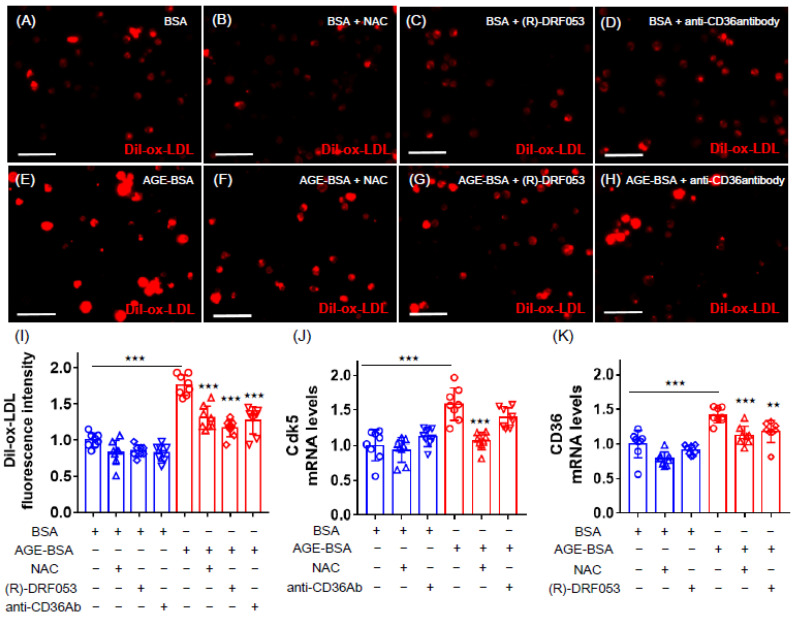
Effects of *N*-acetyl-l-cysteine (NAC), (R)-DRF053 dihydrochloride, and anti-CD36 antibody on Dil-ox-LDL uptake, *Cdk5* and *CD36* gene expression in AGE-exposed U937 cells. U937 macrophages were treated with 100 µg/mL AGE-BSA or 100 µg/mL nonglycated BSA in the presence or absence of 1 mmol/L antioxidant NAC, 0.215 µmol/L selective inhibitor of Cdk5 (R)-DRF053 dihydrochloride, or 10 µg/mL anti-CD36 antibody in RPMI 1640 medium supplemented with 10% fetal bovine serum including 100 ug/mL streptomycin and 100 U/mL penicillin at 37 °C in 5% CO_2_ for 24 h. The cells then were incubated with 10 μg/mL Dil-ox-LDL in the same RPMI 1640 medium for 18 h to evaluate the fluorescence intensity. (**A**–**H**) Representative immunofluorescent staining images. Dil-ox-LDL-positive cells were stained in red. Scale bars represent 50 µm. (**I**) Quantitative data of fluorescence intensity. Dil-ox-LDL uptake was normalized by the control values with BSA. (**J**,**K**) Gene expression levels of *Cdk5* (**J**) and *CD36* (**K**). Total RNAs were transcribed and amplified by real-time PCR. Data were normalized by the intensity of *GAPDH* mRNA-derived signals and then related to the control values with BSA. Number = 8 for each group. Results are presented as mean ± standard deviation. ^★★★^ *p* < 0.005, ^★★^ *p* < 0.01 vs. treatment of AGE-BSA.

We then examined how AGEs increased the Dil-ox-LDL uptake into U937 cells. As shown in Figure 2, an antioxidant, *N*-acetyl-l-cysteine (NAC), mimicked the effects of RAGE-aptamer on AGE-exposed U937 cells; it significantly inhibited the AGE-induced Dil-ox-LDL uptake, as well as *Cdk5* and *CD36* gene expression in macrophages. A selective inhibitor of Cdk5, (R)-DRF053, dihydrochloride attenuated the Dil-ox-LDL uptake and *CD36* gene expression in AGE-exposed U937 cells, whereas anti-CD36 antibody inhibited the Dil-ox-LDL uptake but not *Cdk5* gene expression (Figure 2).

## 3. Discussion

We recently found that AGEs increase the foam cell formation of, and gene expression of *CD36* in, human macrophages [26]. However, the underlying molecular mechanisms remain largely unknown. To address the issue, we first examined the effects of RAGE-aptamer on Dil-ox-LDL uptake, and *Cdk5* and *CD36* gene expression in AGE-exposed human macrophage U937 cells. We found here that RAGE-aptamer significantly suppressed the AGE-induced foam cell formation evaluated by ox-LDL uptake, and *Cdk5* and *CD36* gene expression in U937 cells. We previously showed that RAGE-aptamer at 100 nmol/L, the same concentration used in the present experiments, significantly inhibited the binding of AGEs to RAGE and attenuated oxidative stress and inflammatory reactions in human cultured mesangial cells and melanoma cells, whereas 100 nmol/L control-aptamer did not affect the binding of AGEs to RAGE at all [16,18]. These observations suggest that the AGE-induced foam cell formation and the gene expression of *Cdk5* and *CD36* in U937 cells could be mediated by the interaction with RAGE. In other words, RAGE-aptamer inhibited these harmful effects of AGEs on macrophages by blocking the binding of AGEs to RAGE. Compared with neutralizing antibodies or peptides, aptamers have less immunogenicity and are thermally stable [16,18,36]. Moreover, aptamers can more efficiently penetrate into various tissues over antibodies due to their small size, their pharmacokinetics can be easily improved by chemical modification, and their production costs are low [18,36]. As macrophage foam cell formation is one of the crucial elements of atherosclerosis [20] and the concentration of AGE-BSA used in the present experiments (100 µg/mL) is comparable with that of an in vivo-diabetic situation [2,26], our present study suggests that inhibition of the AGE–RAGE axis by RAGE-aptamer in macrophages may attenuate the progression of atherosclerosis in vivo.

AGEs exert deleterious effects on numerous types of cells through the interaction with RAGE via intracellular oxidative stress generation [37,38,39,40]. In this study, we found that an antioxidant, NAC, mimicked the beneficial effects of RAGE-aptamer on U937 cells; NAC inhibited Dil-ox-LDL uptake into, and *Cdk5* and *CD36* mRNA up-regulation in, AGE-exposed U937 cells, whereas NAC alone did not affect these parameters in nonglycated BSA-treated cells. These observations suggest that the AGE-RAGE-induced oxidative stress could up-regulate *Cdk5* and *CD36* mRNA levels in, and induce foam cell formation of, U937 macrophages.

**Figure 3:**
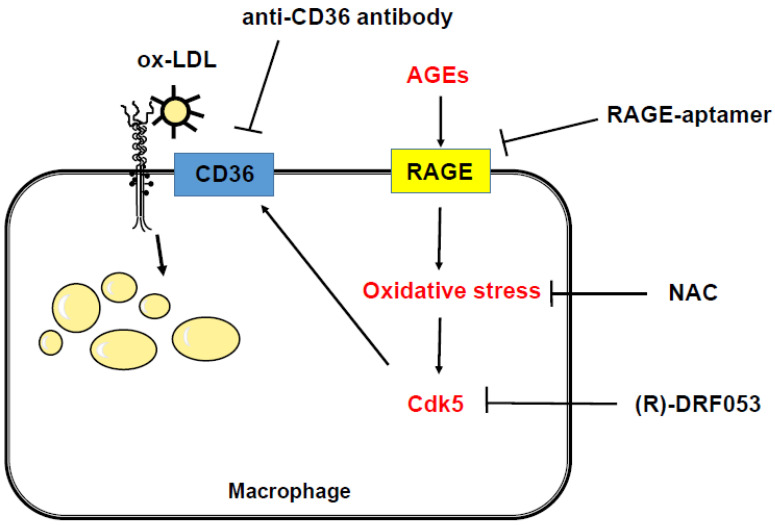
Possible mechanisms for AGE stimulation of the foam cell formation in macrophages. AGE-RAGE-induced oxidative stress generation may stimulate macrophage foam cell formation through the transcriptional induction of CD36 via the Cdk5 pathway. RAGE, receptor of AGE; Cdk5, cyclin-dependent kinase 5; NAC, *N*-acetyl-l-cysteine; ox-LDL, oxidized low-density lipoprotein.

In the present study, gene expression levels of *Cdk5* and *CD36* in AGE-exposed U937 cells were correlated with each other. CD36 is one of the main scavenger receptors that can mediate ox-LDL uptake into macrophages [20], whereas Cdk5 has been shown to contribute to inflammatory reactions in macrophages [30]. Therefore, we next investigated the involvement of Cdk5 and CD36 in AGE-induced foam cell formation using various types of inhibitors. To the best of our knowledge, we are the first to find that an inhibitor of Cdk5 significantly blocked the AGE-induced *CD36* gene expression and Dil-ox-LDL uptake, while an anti-CD36 antibody inhibited the uptake of Dil-ox-LDL into macrophages. In addition, the *Cdk5* gene expression levels were not significantly suppressed by the treatment with anti-CD36 antibody. These findings suggest that CD36 could be mainly involved in AGE-induced foam ell formation and that AGE-RAGE-mediated oxidative stress generation may up-regulate *CD36* mRNA levels in U937 cells via *Cdk5* gene expression. Oxidative stress is reported not only to induce the translocation of Cdk5 from cytoplasm to the nucleus, which could alter its substrate specificity [41], but also to increase the Cdk5 activity in experimental motor neuron disease [42]. Cdk5 has been shown to induce the phosphorylation of peroxisome proliferator-activated receptor γ (PPARγ) and, subsequently, to stimulate its transcriptional activity, being involved in adipogenesis and insulin resistance [43,44]. In addition, PPARγ is known to stimulate macrophage foam cell formation via the transcriptional induction of CD36 [45,46]. Since CD36 expression is enhanced by oxidative stress via PPARγ [47,48], our present study suggests that AGE-RAGE-induced oxidative stress generation may stimulate macrophage foam cell formation through the transcriptional induction of CD36 via the Cdk5–PPARγ pathway (Figure 3). Further studies are needed to clarify whether RAGE-aptamer could attenuate the development and progression of experimental atherosclerosis in animal models by inhibiting macrophage foam cell formation via the suppression of Cdk5-CD36 pathway.

The present study has some potential limitations. First, we previously showed that RAGE-aptamer alone does not have nonspecific effects on mesangial cells, endothelial cells, and melanoma cells [16,18]. In addition, we found here that, when compared with control-aptamer, RAGE-aptamer significantly inhibited the AGE-induced Dil-ox-LDL uptake, and *Cdk5* and *CD36* gene expression in macrophages. Therefore, it is unlikely that RAGE-aptamer has off-target effects on macrophages. Second, to justify the 24-h exposure experiments with AGEs, it would be helpful to examine the time-dependent effects of AGEs on macrophages. Third, although an antioxidant, NAC, inhibited the harmful effects of AGEs on U937 cells, it would also be interesting to measure intracellular oxidative stress in AGE-exposed macrophages. Fourth, we here examined the effects of AGEs on macrophage foam cell formation focusing only on ox-LDL uptake, and *Cdk5* and *CD36* gene expression in vitro. It remains unclear whether continuous administration of RAGE-aptamer or NAC could inhibit foam cell formation within the atherosclerotic plaques of animal models via the inhibition of the Cdk5-Cd36 pathway. Fifth, in this study, we evaluated *CD36* gene expression with quantitative real-time RT-PCR using a standard curve. Although we did not examine protein expression levels of CD36 in the present experiments, we found here that *CD36* gene expression and Dil-ox-LDL uptake in U937 cells were correlated with each other, and that both were significantly inhibited by the treatment with RAGE-aptamer or NAC. As protein expression levels of CD36 are functionally correlated with ox-LDL uptake of macrophages [20], gene expression of *CD36* could reflect the cell surface expression of this protein. In addition, the activity of Cdk5 was reported to be highly correlated with that of *Cdk5* mRNA [28,49]. In the present study, we found that (R)-DRF053 dihydrochloride, a selective inhibitor of Cdk5, blocked the AGE-induced foam cell formation and *CD36* gene expression levels of macrophages. These observations suggest the involvement of the CD36-Cdk5 pathway in ox-LDL uptake into macrophages. Sixth, the magnitude of decrease in CD36 gene expression in U937 cells by various inhibitors was similar to that in Dil-ox-LDL uptake. This observation suggests that CD36 may be the main receptor that could mediate the ox-LDL uptake into macrophages. However, it would be interesting to assess the effects of RAGE-aptamer on other scavenger receptors and cholesterol efflux. Furthermore, experiments with lentiviral or retroviral knockdown of CD36 could prove our speculation. Seventh, we did not examine the effects of AGEs on the immunophenotype change of macrophages. Such information would improve the present works. Eighth, it would also be helpful to investigate whether AGEs could stimulate macrophage foam cell formation through the transcriptional activation of *CD36* gene via the Cdk5-induced activation of PPARγ and if RAGE-aptamer could prevent the progression of atherosclerosis in diabetic animals by suppressing the Cdk5–PPARγ–CD36 pathway.

## 4. Materials and Methods

### 4.1. Materials

BSA and d-glyceraldehyde were purchased from Sigma-Aldrich (St. Louis, MO, USA). An antioxidant, NAC, and anti-CD36 antibody were purchased from Abcam (ab143032 and ab23680, respectively, Cambridge, UK). A selective Cdk5 inhibitor, (R)-DRF053, was obtained from R&D System, Inc. (Minneapolis, MN, USA). A human monocytic cell line, U 937 cells (JCRB9021), was purchased from Japanese Collection of Research Bioresources (JCRB, Osaka, Japan). Dil-ox-LDL was obtained from Highland Technology Center (Frederick, MD, USA). Phorbol 12-myristate 13-acetate was purchased from Sigma Aldrich (St. Louis, MO, USA).

### 4.2. Preparation of AGE-BSA

BSA (25 mg/mL) was incubated under sterile conditions with 0.1 mol/L d-glyceraldehyde in 0.2 M NaPO_4_ buffer (pH 7.4) at 37 °C for 7 days. Then, unincorporated sugars were removed by dialysis against phosphate-buffered saline, as described previously [26,50]. Control nonglycated BSA was incubated in the same conditions except for the absence of D-glyceraldehyde. The extent of lysine modification of AGE-BSA was 65% as described previously [51].

### 4.3. Preparation and Selection of RAGE-Aptamer

A random combinatorial single-stranded DNA library with normal phosphate ester backbone oligonucleotides (80 mer) was synthesized, and a RAGE-aptamer was selected using systematic evolution of ligands by exponential enrichment (SELEX) method as described previously [16,17,52,53]. Sequences of the selected RAGE-aptamer and the control-aptamer were as follows: RAGE-aptamer; 5′-CCTGATATGGTGTCACCGCCGCCTTAGTATTGGTGTCTAC-3′, control-aptamer; 5′-ATCGACCTGGAGGCGAGCAGCTCGGATCCAGTCGCGTGAG-3′. The RAGE-aptamer and control-aptamer were modified with phosphorothioate as described previously [16,17,52,53].

### 4.4. Experiments of U937 Macrophages

U937 cells were maintained in Roswell Park Memorial Institute (RPMI) 1640 medium with 10% fetal bovine serum including 100 μg/mL streptomycin and 100 U/mL penicillin. The cells were seeded onto 3.5 cm dishes (1.0 × 10^6^ cells/dish) and incubated with phorbol 12-myristate 13-acetate (40 ng/mL) in RPMI 1640 medium supplemented with 10% fetal bovine serum at 37 °C in 5% CO_2_ for 24 h. After gentle rinsing with phosphate-buffered saline (PBS) three times, adherent cells were used as differentiated macrophages. In previous reports, fluorescence-activated cell sorting (FACS) analysis showed that the adherent cells prepared similarly were differentiated macrophages [26,54,55,56,57]. U937 macrophages were treated with 100 µg/mL AGE-BSA or 100 µg/mL nonglycated BSA in the presence or absence of 100 nmol/L RAGE-aptamer, 100 nmol/L control-aptamer [16], 1 mmol/L NAC, 0.215 µmol/L (R)-DRF053 dihydrochloride, or 10 µg/mL anti-CD36 antibody in RPMI 1640 medium supplemented with 10% fetal bovine serum including 100 µg/mL streptomycin and 100 U/mL penicillin at 37 °C in 5% CO_2_ for 24 h. After completion of the incubation, the U937 macrophages were used for immunohistochemistry or real-time reverse-transcription polymerase chain (RT-PCR) analyses.

### 4.5. Uptake of Dil-ox-LDL into Macrophages

The U937 macrophages were incubated in the presence of 10 μg/mL Dil-ox-LDL in RPMI 1640 medium supplemented with 10% fetal bovine serum including 100 μg/mL streptomycin and 100 U/mL penicillin at 37 °C in 5% CO_2_ for 18 h [21,26]. After gentle washing with PBS three times, the adherent cells were mounted in Vectashield mounting medium (H-1500, Vector Laboratories, Burlingame, CA, USA) and were imaged with BZ-X710 microscope/software (Keyence, Osaka, Japan). The fluorescent intensity of red color area per cells was quantified as described previously [21,26].

### 4.6. Gene Expression Levels

The total RNA was isolated with QIAGEN reagents (Hilden, Germany), and the gene expression was analyzed by real-time RT-PCR using the gene expression assay of SYBR Green or TaqMan, and a sequence detection system (ABI PRISM 7900, Life Technologies, Palo Alto, CA, USA) as described previously [21,22,23,26,58]. Values were normalized by the intensity of glyceraldehyde 3-phosphate dehydrogenase (*GAPDH)* mRNA-derived signals and then related to the control values with BSA. Primers and probes were as follows: *Cdk5*, NM_001164410.3, NM_004935.4; *CD36*, Hs00169627_ml; *Gapdh*, Hs99999905_ml.

### 4.7. Statistical Analysis

Values are shown as mean ± standard deviation. Analysis of variance (ANOVA) was used for statistical comparison among multiple groups. Pearson’s correlation test was performed to examine the correlation between two groups. All statistical analyses were performed by PRISM 7.0 software (GraphPad, San Diego, CA, USA), and *p* < 0.05 was defined as statistically significant.

## 5. Conclusions

The present study is the first to show that the AGE-RAGE axis could stimulate ox-LDL uptake into macrophages via the oxidative stress-Cdk5-CD36 pathway. Inhibition of the AGE-RAGE axis in macrophages may be a novel therapeutic target for the management of accelerated atherosclerosis in diabetes mellitus.
